# Genome Sequence of *Salmonella bongori* Strain N268-08, a Rare Clinical Isolate

**DOI:** 10.1128/genomeA.00580-13

**Published:** 2013-08-22

**Authors:** Roger Marti, Steven Hagens, Martin J. Loessner, Jochen Klumpp

**Affiliations:** Institute of Food, Nutrition and Health, ETH Zürich, Zürich, Switzerlanda; Micreos Food Safety, Wageningen, The Netherlandsb

## Abstract

*Salmonella bongori* is a close relative of the highly virulent members of *S. enterica* subspecies *enterica*, encompassing more than 2,500 serovars, most of which cause human salmonellosis, one of the leading food-borne illnesses. *S. bongori* is only very rarely implicated in infections. We here present the sequence of a clinical isolate from Switzerland, *S. bongori* strain N268-08.

## GENOME ANNOUNCEMENT

The complete genome sequence of *Salmonella bongori* strain N268-08 was determined *de novo* using the Pacific Biosciences SMRT sequencing technology (Functional Genomics Center, Zurich, Switzerland). Assembly was carried out using the SMRT-Analysis software (version 2.0, HGAP assembly protocol), and the sequence was automatically annotated using RAST (http://rast.nmpdr.org/) (1). It was aligned and compared to that of *S. bongori* NCTC12419 (2) by progressive Mauve (3, 4).

Sequence information for N268-08 is provided in comparison to NCTC12419 (2). The sequence of N268-08 is 4.7 Mbp (NCTC12419, 4.5 Mbp) long, and the G+C content is 51.3% (NCTC12419, 51.3%). A total of 4,642 coding sequences (CDS) (NCTC12419, 4,054 CDS), 22 rRNAs (NCTC12419, 22 rRNAs), and 86 tRNAs (NCTC12419, 84 tRNAs) were identified. Mauve alignment revealed that a prophage (~30 kb) seemed to be inverted and inserted at another location in N268-08 (2,769,647 to 2,738,043 versus 3,084,741 to 3,115,162 in NCTC12419).

N268-08 features two major deletions (>20 kb) compared to NCTC12419, in a 23.2-kb prophage (304,635 to 327,860) and a 27.6-kb region (2,013,350 to 2,040,915) encoding two putative phage integrases, transcriptional regulators, and membrane transport proteins. N268-08 features 6 insertions (>20 kb). Four are putative prophages: 37.8 kb (478,064 to 515,915) with similarities to phage Mu; 20.0 kb (1,022,461 to 1,042,491); 55.3 kb (1,317,949 to 1,373,269); and 50.8 kb (1,985,799 to 2,036,593) with two Gifsy-2-like proteins. The other insertions are 21.7 kb (1,721,254 to 1,742,938) with hypothetical proteins and 62.2 kb (4,528,603 to 4,590,835) featuring genes associated with fimbriae and type IV secretory-pathway-associated proteins VirB4/D4 (5, 6).

*De novo* assembly yielded a 90.0-kb plasmid featuring the *tra* operon of incompatibility group F. The best hits (BLASTn) are to *Salmonella* plasmids p14-120 (accession no. JQ418538) and pSGSC3045-121 (accession no. JQ418541).

The sequence type (as proposed in reference 7) of N268-08 is novel (*aroC132* *dnaN30* *hemD103* *hisD134* *purE126* *sucA133* *thrA129*) (http://mlst.ucc.ie/mlst/mlst/dbs/Senterica/).

### Nucleotide sequence accession numbers. 

The genome and plasmid sequences of N268-08 have been deposited under the accession numbers CP006608 and CP006609 at NCBI.
