# Genome Sequence of *Salmonella bongori* Strain N268-08

**DOI:** 10.1128/genomeA.01018-13

**Published:** 2013-12-12

**Authors:** Roger Marti, Steven Hagens, Martin J. Loessner, Jochen Klumpp

**Affiliations:** Institute of Food, Nutrition and Health, ETH Zürich, Zürich, Switzerland; Micreos Food Safety, Wageningen, The Netherlands

## AUTHOR CORRECTION

Volume 1, no. 4, e00580-13, 2013. Page 1: The article title should read as given above. After the publication of this article, we were made aware of the fact that strain N268-08 was not isolated from a clinical sample.

